# Effects of short‐ and long‐term plant functional group loss on alpine meadow community structure and soil nutrients

**DOI:** 10.1002/ece3.10919

**Published:** 2024-03-11

**Authors:** Jingjing Wei, Huakun Zhou, Xinqing Shao, Jian Sun, Li Ma, Zhonghua Zhang, Ruimin Qin, Hongye Su, Xue Hu, Tao Chang, Zhengchen Shi, Haze Ade, Huichun Wang

**Affiliations:** ^1^ College of Geographical Science Qinghai Normal University Xining China; ^2^ Qinghai Provincial Key Laboratory of Restoration Ecology in Cold Regions, Northwest Institute of Plateau Biology Chinese Academy of Sciences Xining China; ^3^ State Key Laboratory of Tibetan Plateau Earth System, Environment and Resources (TPESER), Institute of Tibetan Plateau Research Chinese Academy of Sciences Beijing China; ^4^ China Agricultural University Beijing China; ^5^ University of Chinese Academy of Sciences Beijing China

**Keywords:** community cohesion, community structure, plant functional groups loss, soil properties, species diversity

## Abstract

The rapid loss of global biodiversity can greatly affect the normal functioning of ecosystems. However, how biodiversity losses affect plant community structure and soil nutrients is unclear. We conducted a field experiment to examine the short‐ and long‐term effects of removing plant functional groups (Gramineae, Cyperaceae, legumes, and forbs) on the interrelationships among the species diversity, productivity, community structure, and soil nutrients in an alpine meadow ecosystem at Menyuan County, Qinghai Province. The variations in the species richness, above‐ and belowground biomass of the community gradually decreased over time. Species richness and productivity were positively correlated, and this correlation tended to be increasingly significant over time. Removal of the Cyperaceae, legumes, and other forbs resulted in fewer Gramineae species in the community. Soil total nitrogen, phosphorus, organic matter, and moisture contents increased significantly in the legume removal treatment. The removal of other forbs led to the lowest negative cohesion values, suggesting that this community may have difficulty recovering its previous equilibrium state within a short time. The effects of species removal on the ecosystem were likely influenced by the species structure and composition within the community. Changes in the number of Gramineae species indicated that they were more sensitive and less resistant to plant functional group removal. Legume removal may also indirectly cause distinct community responses through starvation and compensation effects. In summary, species loss at the community level led to extensive species niche shifts, which caused community resource redistribution and significant changes in community structure.

## Graphical abstract

Gramineae are less resistant to removal behavior. The removal of diverse plant functional groups resulted in differences in the compensatory effects of the remaining vegetation. Complete removal of the Forb would make it difficult for the community to return to its previous equilibrium in a short time. Changes in species niches would affect community succession trends.

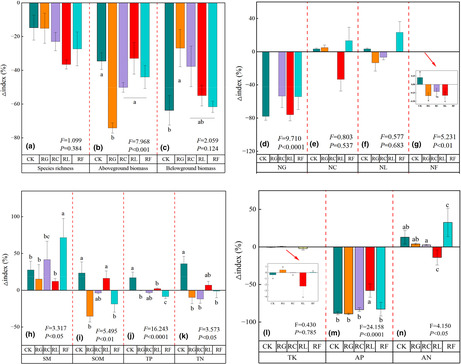

## 1 INTRODUCTION

Anthropogenic stressors such as global climate change, bioinvasion, and land abuse are serious threats to terrestrial ecosystems worldwide that have led to the rapid extinction of some species in terrestrial ecosystems (Sala et al., 2000). Over the last several decades, many studies have attempted to determine the potential impacts of dramatic reductions in biodiversity on ecosystem functions and services (Cardinale et al., 2012). Some studies have demonstrated that species diversity loss can severely influence plant community features, such as primary productivity and plant interrelationships, and these impacts frequently determine the characteristics of the remaining plant functional groups (PFGs) (Balvanera et al., 2006; Cardinale et al., 2012). Species could generally be categorized into resource‐acquiring species and resource‐conserving species. For example, resource‐acquiring species are more likely to persist in a wide range of environments, whereas resource‐conserving species have the greatest influence on less productive ecosystems (Streit et al., 2022). The removal of different PFGs can also affect ecosystem processes, structural, and functional responses of belowground communities, including carbon content and soil community composition (Cardinale et al., 2012; Chen et al., 2022). Many studies attribute the effects of species loss on belowground communities to the impact of aboveground plant biomass on the quality and quantity of resources for belowground community (Gastine et al., 2003; Milcu et al., 2008). Because plant biomass is the primary source of carbon and energy for belowground communities (Wardle et al., 2004), litter deposition also modifies plant biodiversity, species composition, and ecosystem processes, which are influenced by changes in species loss or species relative abundance (Ladouceur et al., 2022). Global extinction rates are not effectively represented by local‐scale estimates of species richness and diversity (Blowes et al., 2019; Dornelas et al., 2014). Therefore, the understanding of the effects of PFG declines on local‐scale above‐ and belowground communities remains limited.

PFG loss affects the soil biome by reducing plant biomass and influencing resource inputs through plant apoplast and rhizome deposition (hereafter referred to as the energy starvation mechanism) (Fanin et al., 2019). Species loss may influence soil biota by reducing plant biomass. Litter decomposition could profoundly affect critical processes such as soil organic matter formation, nutrient cycling, energy flow, and plant growth. In addition, litter decomposition may directly or indirectly affect plant–plant interactions, community succession, microbial diversity and activity, and biodiversity–ecosystem function (BEF) relationships (Zhang et al., 2023). All species contribute uniquely to determining ecosystem function (hereafter referred to as the niche complementarity mechanism). The niche complementarity hypothesis assumes that species‐rich communities can more efficiently access and utilize limited resources because they contain species with a variety of ecological attributes. The ecosystem is considered to be more functionally complete as these species complement each other, enabling them to optimize resource use (Firn et al., 2007). However, in some studies, only a small number of species within a community have been found to influence ecosystem function, indicating the importance of dominant or keystone species (Engelhardt & Ritchie, 2001; Firn et al., 2007). Although the energy starvation and niche complementarity mechanisms may individually or together determine the impact of PFGs on soil structure and soil community function, their relative contributions require further investigation. Some studies have indicated that PFG removal dramatically affected above‐ and belowground communities and their dominant ecosystem processes (Zhang et al., 2017). Moreover, species diversity appears to regulate plant–soil relationships and plant growth strategies at different stages, which in turn influence the processes involved in plant niche differentiation (Peter et al., 2022). The speed at which species adjust to diversity loss and the subsequent impact on local‐scale community shifts also remain unknown.

Therefore, we explored two nonmutually exclusive mechanisms (energy starvation and niche complementarity) that may explain the effects of PFG loss on community structure and nutrient cycling by experimentally removing four PFGs (Gramineae, Cyperaceae, legumes, and other forbs) from vegetation communities to simulate diversity loss in an alpine meadow ecosystem on the eastern Qinghai–Tibet Plateau. The four target PFGs were selected based on eco‐physiological traits potentially associated with response variables such as biomass production, resource use patterns, and nitrogen (N) fixation ability (Gross et al., 2007; McLaren & Turkington, 2010; Reich et al., 2001). Cyperaceae species are dominant among alpine vegetation in the study region. Gramineae species conserve resources through their high C:N ratio and leaf dry matter content. Legumes and other forbs promote species and functional diversity, offering different plant resource utilization pathways that enhance nutrient use and release, leading to higher N and P content in alpine plant communities (Chen et al., 2022). We separately analyzed data for treatment periods of 3 years (2012–2015) and 10 years (2012–2022) to compare short‐ and long‐term effects of PFG loss. Our objectives were to: (1) evaluate the effects of PFG removal on species diversity, community composition structure, and soil properties; investigate differences between short‐ and long‐term effects; (2) determine the key environmental factors influencing above‐ and belowground development following species loss.

**FIGURE 1:**
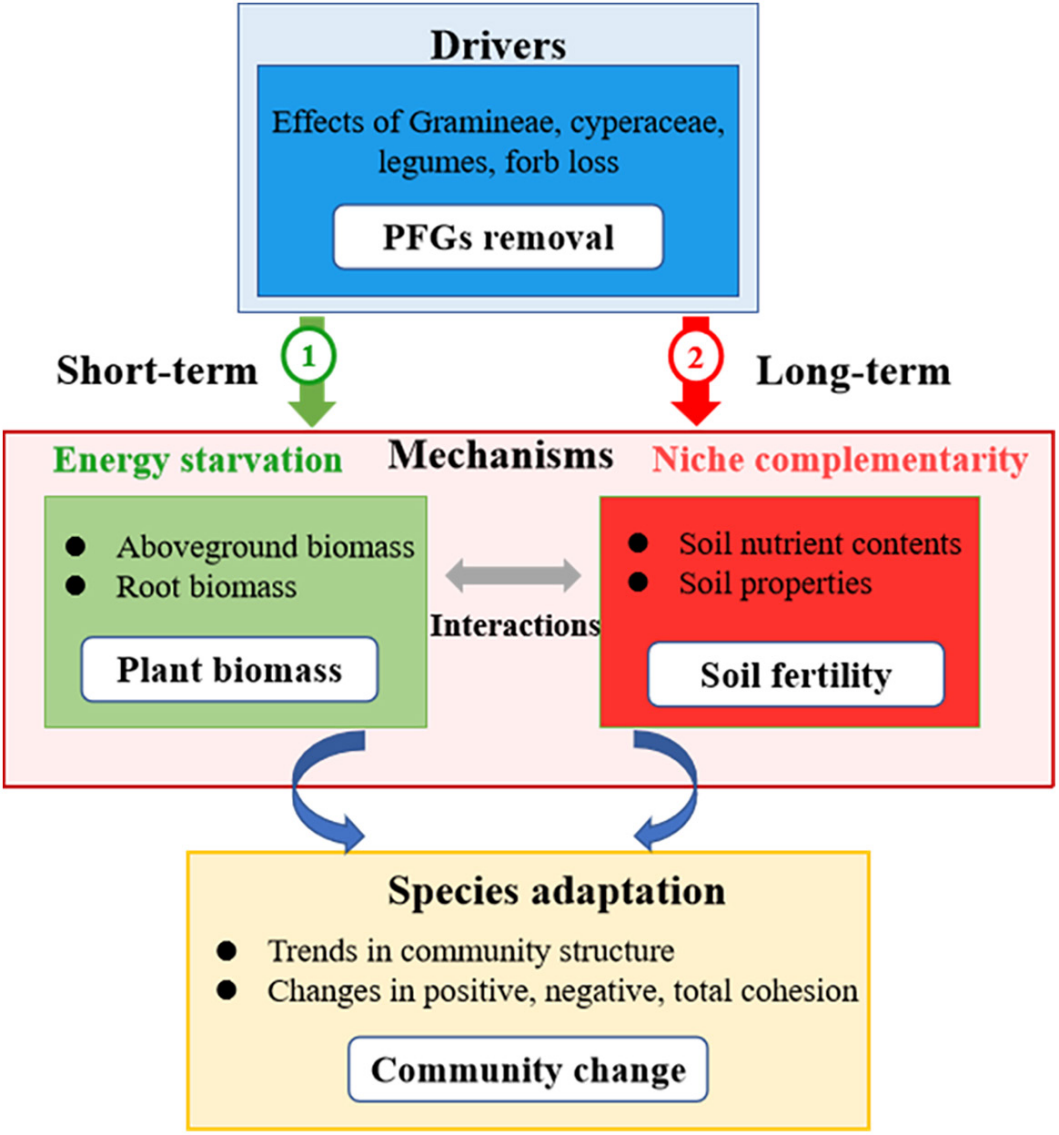
Schematic representation of the effect of plant functional groups loss (PFGs, blue box) on community change (yellow box) when each of the two mechanisms is considered, thus changing cohesion among community. Mechanism 1 (green box, “Energy starvation” hypothesis); Mechanism 2 (red box, “Niche complementarity” hypothesis): PFG loss effect trend in community structure, thereby influence soil nutrient and properties).

These experiments were conducted to test two hypotheses. First, the energy starvation mechanism during short‐term species loss is likely to be the primary driver influencing plant community structure, biomass, and soil nutrients, then the impact of PFG removal on soil nutrients will depend on community biomass input and productivity (Chen et al., 2016; Ward et al., 2009). Because community biomass must decrease with PFG removal, we assumed that removal of the target PFG would greatly reduce both litter fall and soil organic matter content (Figure 1). Litter decomposition may affect the soil nutrient enrichment, soil biodiversity, and species diversity. Greater plant species diversity significantly may influence the quantity, quality, and chemical diversity of plant litter (Kou et al., 2020; Porre et al., 2020). Second, niche complementarity mechanism may be the dominant driver of changes in community structure during long‐term species loss, then the impact of PFG removal on community stability will depend on changes in soil nutrients and plant community characteristics (Figure 1). Species richness is explained in terms of stable coexistence attributable to properties of the local environment, the niche of each species is different and these differences collectively stabilize community dynamics. Niche stabilizes diversity at some fixed level and provides a floor on species richness where immigration is low, but the community niche balance will be changed dynamically as PFGs are removed from the community (Loke & Chisholm, 2023). Under these conditions, community compositional shifts are limited and equilibrium is more likely to be restored after an environmental perturbation (Agler et al., 2016; Coyte et al., 2015; Herren & McMahon, 2017). Therefore, we hypothesized that the long‐term removal of PFGs would cause niche divergence among the remaining species in the community, as well as resource redistribution of resources that would affect community resilience. Our results help elucidate the influence of PFG loss on the aboveground communities and soil nutrient cycling, and enhance the understanding of species adaptations under short‐ and long‐term species loss at the local scale.

## 2 METHODS

### 2.1 Study site

**FIGURE 2:**
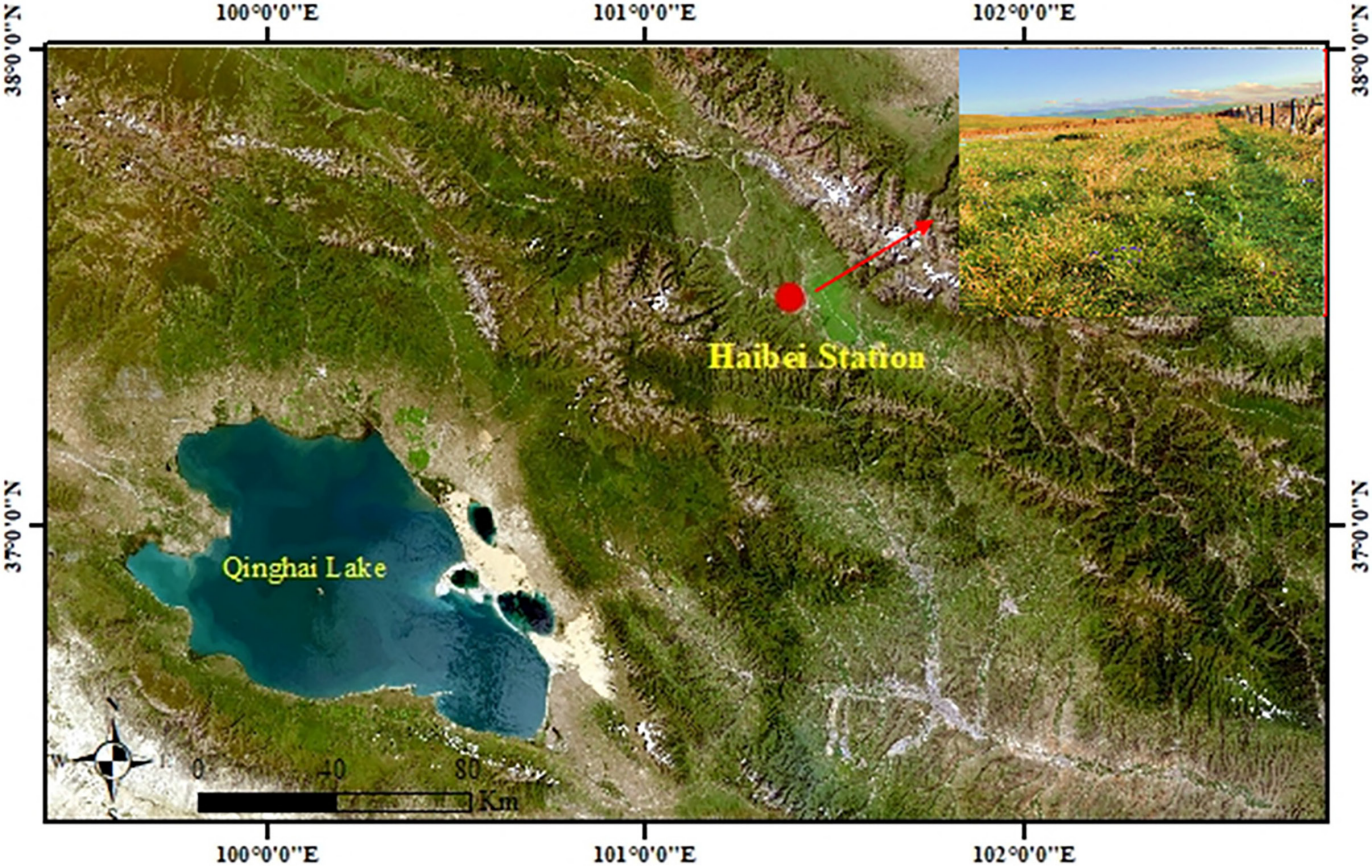
Study area location and site characteristics (Satellite layer data source: http://www.locaspace.cn/).

The experiments were conducted at the Haibei Alpine Meadow Ecosystem Research Station (37°29′–37°45′ N, 101°12′–101°23′ E; 3200–3600 m), which located at Menyuan County, Qinghai Province, China (Figure 2). The study area has a continental highland climate, with a mean average temperature of −1.7°C and mean annual precipitation of 580 mm (Chen et al., 2022). The vegetation is typical of a subalpine meadow, dominated by Cyperaceae and diverse forbs. Gramineae and legumes are rare. The most abundant plant species are *Carex alatauensis* and *Carex parva* (Cyperaceae), accounting for 40% of the total coverage. The soil layers are young and shallow, characterized by simple formation processes; the soil is weakly alkaline and poor in plant‐available nutrients but rich in immobile nutrients (Zhang et al., 2017).

### 2.2 Experimental design

The experiment was conducted on a flat, undisturbed site that was established in 2012. For conservation purposes and to enable regular experimentation, the site was fenced off. The experiment design had a completely randomized design and involved complete removal of 0–4 PFGs. Five treatments were set up, including the control (CK), removal of Gramineae (RG), removal of Cyperaceae (RC), removal of legumes (RL), and removal of forbs (RF). Each plot was attributed to a treatment. Each of the five treatment plots (1 m × 0.75 m) was replicated five times, in a randomized design with 1 m spacing between plots. The aboveground and belowground parts (stems, leaves, and roots) of the targeted PFGs were cut twice per month from 2012 to 2022, until the plants were completely cleared, to prevent their growth while minimizing repeated physical disturbance to the soil (Chen et al.,^2016^; Naeem,^2002^). At the peak of vegetative biomass (August), we recorded the species composition, frequency, cover, height, and soil nutrients within each plot in 2015 and 2022 to examine short‐ and long‐term effects, respectively.

### 2.3 Plant sampling and analysis

The experiment began in 2012. In late August 2015 and 2022, the aboveground plant biomass was measured by clipping all plants in a 0.50 m × 0.50 m square at the soil surface in each plot. From each plot, two soil cores (diameter 5 cm; depth 10 cm) were collected and carefully washed to obtain fine root samples as belowground biomass. Above‐ and belowground biomass was measured as dry weight in a drying oven set at 65°C for 48 h (Herren & McMahon, 2017). All species were classified as Gramineae, Cyperaceae, legumes, and other forbs.

Plant diversity was estimated according to species richness and the Margalef richness index, using the following formula (Wu et al., 2012):

(1) $$\;\mathrm{Ma}=\left(S-1\right)/\ln N$$

where *S* is the total number of species and *N* is the total density of all plants in the sample.

The Shannon–Wiener diversity index was accounted as (Wu et al., 2012):

(2) $$\;H=-\sum_{i=1}^{s}\left(P_{i}\ln P_{i}\right)$$

where *P*_*i*_ is the proportion of the *i* species in the sample.

The Simpson dominance index was calculated as (Wu et al., 2012):

(3) $$D=1-\sum_{i=1}^{s}P_{i}^{2}$$

The Pielou evenness index was calculated as (Wu et al., 2012):

(4) E=H/lnS

To gain further insight into the effects of species loss on plant communities, we evaluated positive and negative effects in 2015 and 2022, separately. Cohesion provides insight into interspecies associations that are driven by both positive and negative species interactions and/or by the similarity and variability of each species niche (Herren & McMahon, 2017). We calculated two cohesion values (positive and negative) for each sample *j*, as the sum of significant positive or negative correlations among species, weighted by species relative abundance, as follows:

Positive Cohesion:

(5) $$C_{j}^{\mathrm{pos}}=\sum_{i=1}^{n}a_{i}\cdot {\bar{r}}_{i,r>0}$$

Negative Cohesion:

(6) $$C_{j}^{\mathrm{neg}}=\sum_{i=1}^{n}a_{i}\cdot {\bar{r}}_{i,r<0}$$

where $a_{i}$ is the relative abundance of the *i* ^th^ species in sample *j*, ${\bar{r}}_{i,r>0}$ and ${\bar{r}}_{i,r<0}$ are positive and negative connectedness values, respectively (Herren & McMahon, 2017). Within a specific network (i.e., one of the four PFGs), the positive and negative connectedness for species *i* were calculated as the average of all of its significant positive or negative correlations with all other community species found in the network. Higher absolute values of average negative or positive cohesion indicate greater relevance within the community. Communities with larger negative cohesion values tend to be more stable, as their composition changes less over time (Herren & McMahon, 2017).

### 2.4 Soil sampling and analysis

We analyzed the short‐ and long‐term effects of PFG removal on ecosystem properties using 50 soil samples. Soil moisture content was calculated by oven‐drying the soil samples at 110°C for 10 h. Soil organic matter was determined using the volumetric method, with potassium dichromate. Soil total nitrogen was measured using Kjeldahl method (Bremner, 1996). Soil total phosphorus was extracted using colorimetric analysis (Olsen et al., 1982). Soil total potassium was determined by flame photometry after digestion with HF‐HClO_4_, and soil available phosphorus was determined by the molybdenum‐antimony colorimetric method; soil available nitrogen was measured by the sulfate extraction method (Bao, 2000).

### 2.5 Data processing and analysis

To reduce the effects of inherent spatial heterogeneity among species, soil, and other factors on our results, we analyzed the relative variation in factor indicators (Jiang et al., 2021).

(7) $$∆\text{index}\left(\%\right)=\frac{\left({\text{index}}_{2022}-{\text{index}}_{2015}\right)}{{\text{index}}_{2015}}\times 100$$

where ∆ represents change and index represents any specific analysis index.

Prior to analysis, the data were organized and descriptive statistics were obtained using Excel 2019. The R v4.2.1 statistical platform and Origin 2022 were used for all analyses. To investigate the effects of time and PFG removal type on species richness, plant biomass, community cohesion, soil properties, and species diversity index using a mixed effect model (Table 1; Tables S1 and S2), plot was treated as a random factor, and fixed factors were PFG removal type and time. Differences among treatments under the same year required multiple comparisons to test for variability, and independent samples *t*‐tests were used to test for differences among years for the same treatment.

**TABLE 1:** The *F* and *p* values from the two‐way ANOVA for community cohesion (Positive, Negative, Negative/Positive and Total Cohesion) affected by year and treatment type (CK, RG, RC, RL, and RF).

Source	Positive cohesion			Negative cohesion		Negative/positive cohesion		Total cohesion	
	df	*F*	*p*	*F*	*p*	*F*	*p*	*F*	*p*
Year	1	82.85	<.0001	152.10	<.0001	1.68	.201	238.55	<.0001
Treatment	4	41.04	<.0001	46.97	<.0001	1.38	.258	92.89	<.0001
Year*Treatment	4	24.54	<.0001	42.97	<.0001	19.98	<.0001	43.11	<.0001

Nonmetric multidimensional scaling (NMDS) analysis was used to explore community composition trends after PFG removal by comparing the relative abundance of plants after short‐ and long‐term treatment using the *vegan* package in R. Permutational multivariate analysis of variance (PERMANOVA) was performed using the “adonis” function with 999 permutations in the *ggplot2* and vegan packages in R. Correlation analysis of community composition and environmental variables was performed using the *psych*, *reshape2*, and *ggplot2* packages in R. ArcGIS v10.7 was used to draw a schematic diagram of the sample points (Gweon et al., 2020).

## 3 RESULTS

### 3.1 Response of PFG loss on species and soil properties

**FIGURE 3:**
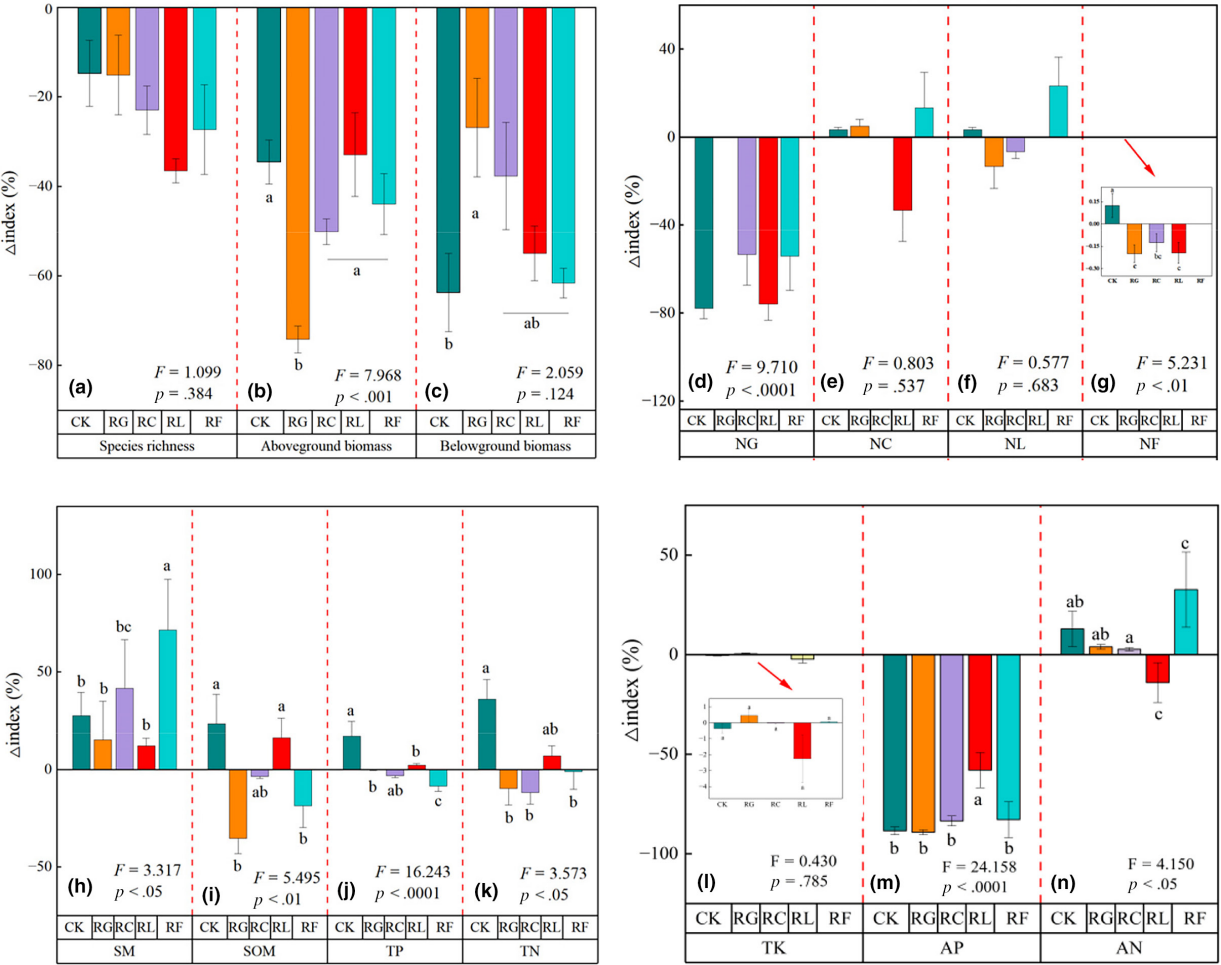
Relative changes in the (a) species richness, (b) aboveground biomass, (c) belowground biomass, (d) number of Gramineae species (NG), (e) number of Cyperaceae species (NC), (f) number of legume species (NL), (g) number of other forb species (NF), (h) soil moisture (SM), (i) soil organic matter (SOM), (j) soil total phosphorus (TP), (k) soil total nitrogen (TN), (l) soil total potassium (TK), (m) soil available phosphorus (AP), (n) soil available nitrogen (AN) of PFG removal treatments between 2015 and 2022. Different letters above or below bars indicate significant differences among PFG removal treatments (*p* < .05; analysis of variance [ANOVA] followed by Duncan's test). Experimental treatments consisted of the removal of Gramineae (RG), Cyperaceae (RC), legumes (RL), other forbs (RF), and the control (CK).

**FIGURE 4:**
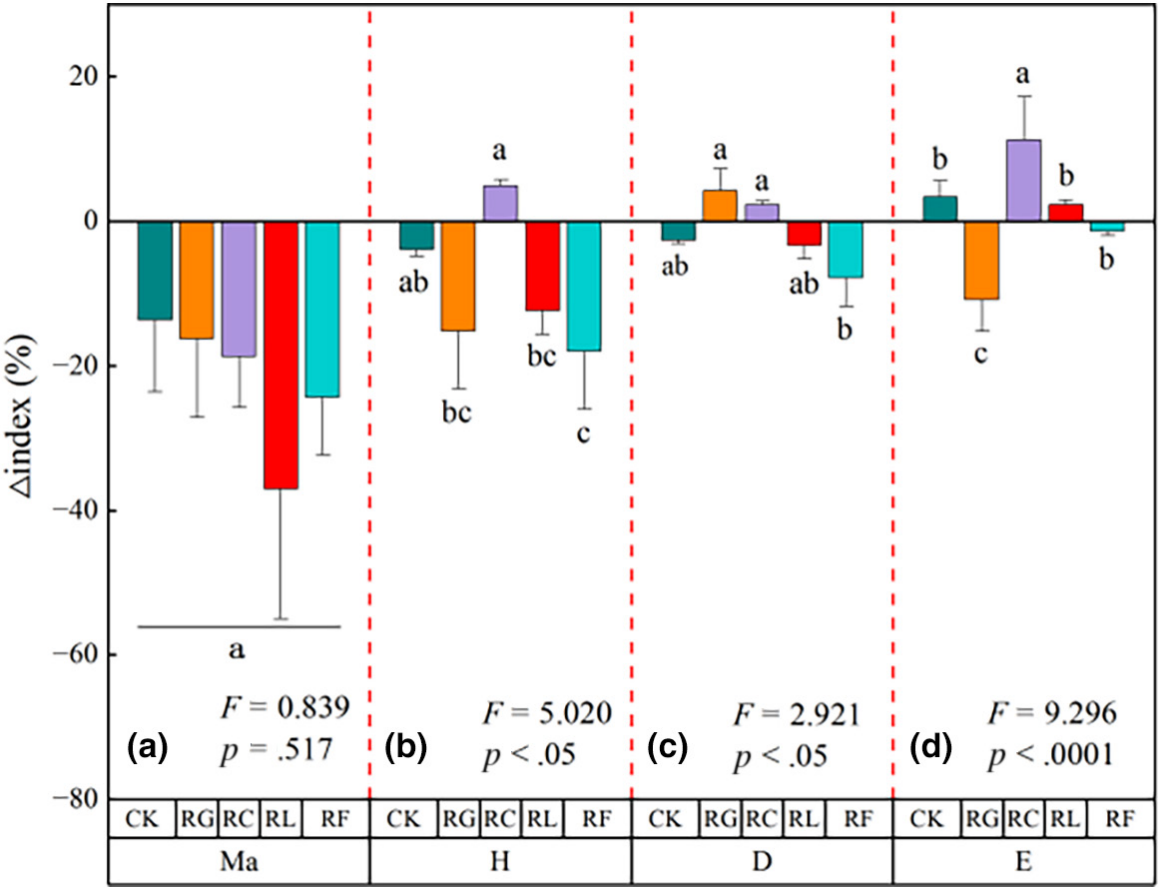
Relative changes in the (a) Margalef richness index (Ma), (b) Shannon–Wiener diversity index (H), (c) Simpson dominance index (D), (d) Pielou evenness index (E) of PFG removal treatments between 2015 and 2022.

Changes in species richness, above‐, and belowground biomass showed a decreasing trend between 2015 and 2022; removal of Gramineae (RG) had a greater impact on reducing aboveground biomass (Figure 3a–c). The number of Gramineae species in the community was significantly reduced in Cyperaceae removal treatment (RC), legume removal treatment (RL), and forb removal treatment (RF) (*p* < .05; Figure 3d–g). Soil moisture content increased significantly with time in all treatments (*p* < .05). Soil nutrient content increased in both RL and CK, yet decreased in all other treatments (Figure 3h–k). Soil total potassium, available phosphorus, and available nitrogen content increased in RL, and no regular changes were observed in the remaining treatments (Figure 3l–n). Species diversity index decreased in RF, and no regular changes in other treatments (Figure 4a–d).

### 3.2 Changes in community cohesion and structure in response to PFG loss

**FIGURE 5:**
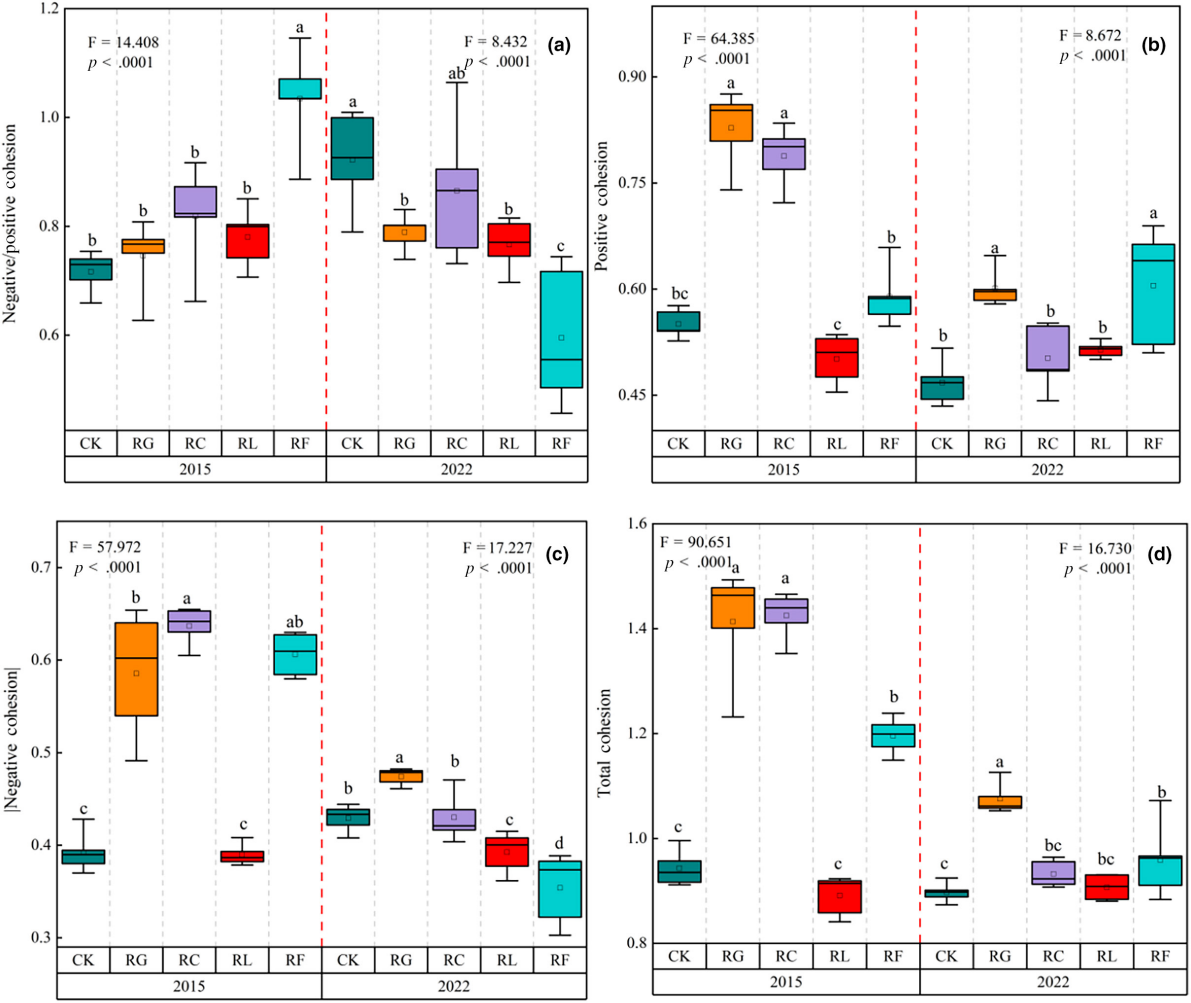
Changes in (a) the ratio of negative to positive cohesion, (b) positive cohesion, (c) negative cohesion, and (d) total cohesion (sum of the absolute values of positive and negative cohesion) in response to PFG loss. Error bars represent 95% confidence intervals. Different letters above bars indicate significant differences between treatments. Red dashed line divides short‐ and long‐term effects.

Positive, negative, and total cohesion values, as well as the ratio of negative to positive cohesion, were all significantly different by PFG removal (*p* < .0001; Figure 5; Table 1). The ratio of negative to positive cohesion was significantly higher following the removal of forbs other than legumes (*p* < .0001; Figure 5a) than in all other treatments in 2015 (i.e., increased negative cohesion; Figure 5c). However, the opposite trend was observed in 2022 (i.e., increased positive cohesion; Figure 5b). The difference in total cohesion was higher in 2015 than in 2022 in all treatments except the legume removal treatment (Figure 5d).

**FIGURE 6:**
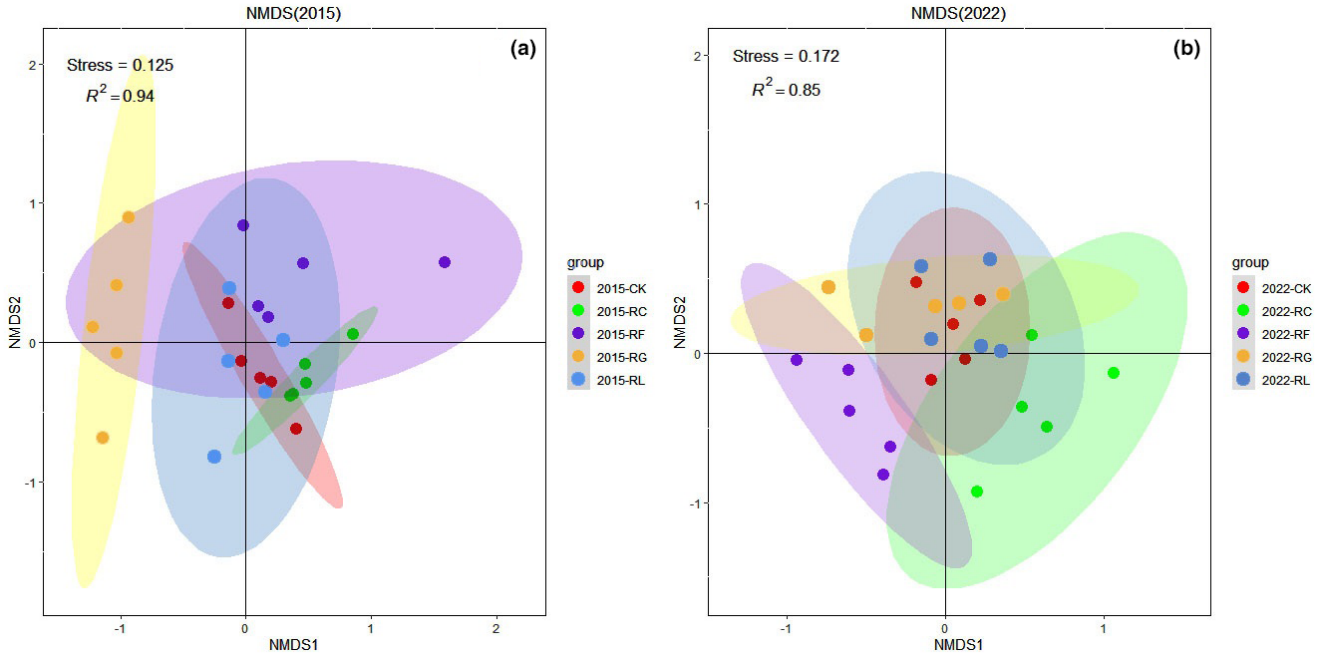
Nonmetric multidimensional scaling (NMDS) analysis based on a Bray–Curtis dissimilarity matrix revealed distinct differences in community composition among all five PFG removal treatments (PERMANOVA; *p* < .005).

The NMDS ranking chart indicated apparent segregation between communities in all five treatments in 2015 and 2022 (PERMANOVA, *p* < .005). The NMDS stress values were 0.125 and 0.172 in 2015 and 2022, respectively, indicating that the fit was an accurate reflection of the actual community characteristics (Figure 6). In 2015, there was greater segregation between the Gramineae removal treatment and all other treatments, although there was slight overlapping between the Gramineae and other forb removal treatments in terms of community composition, and the remaining treatments overlapped both of these treatments. The Cyperaceae removal treatment completely overlapped all other treatments in 2015, indicating a lack of independent community composition (Figure 6a). In 2022, the community composition of each treatment overlapped all other treatments; the Cyperaceae removal treatment showed only partial overlapping with the other treatments, and had a more distinct community composition than observed in 2015 (Figure 6b).

### 3.3 Correlation of species characteristics and soil properties

**FIGURE 7:**
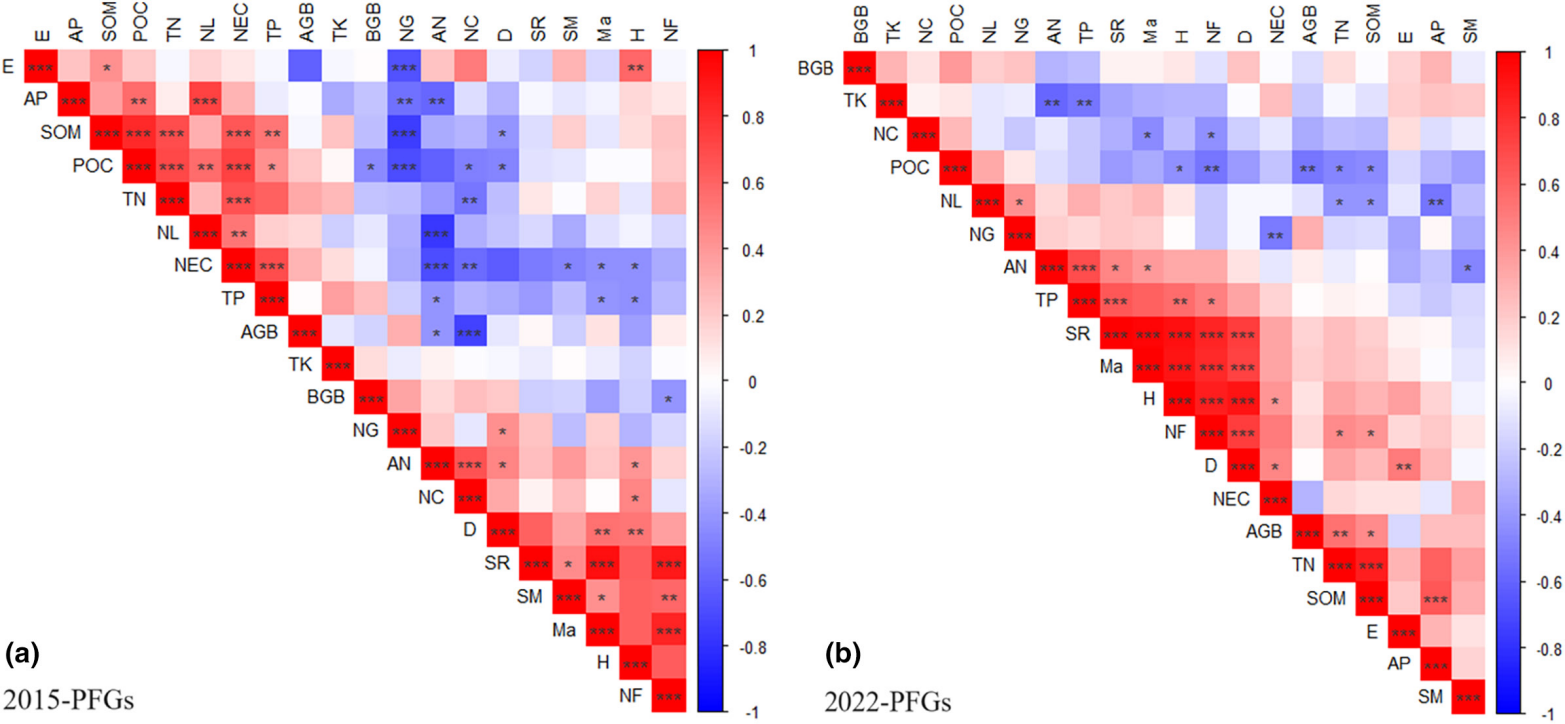
Correlations among plant, soil, and biodiversity variables under PFG loss in (a) 2015 and (b) 2022. Asterisks indicate significance levels (*p < .05; **p < .01; ***p .001). AGB, aboveground biomass; AN, available nitrogen; AP, available phosphorus; BGB, belowground biomass; D, Simpson dominance index; E, Pielou evenness index; H, Shannon–Wiener diversity index; Ma, Margalef richness index; NC, number of Cyperaceae species; NEC, negative cohesion; NF, number of other forb species; NG, number of Gramineae species; NL, number of legume species; POC, positive cohesion; SM, soil moisture; SOM, soil organic matter; SR, species richness; Tk, total potassium; TN, total nitrogen; TP, total phosphorus.

In 2015, there were significant positive correlations between soil organic matter and total nitrogen, available nitrogen, and the number of Cyperaceae species (*p* < .001; Figure 7a). Aboveground biomass was highly significantly negatively correlated with the number of Cyperaceae species. Soil organic matter and the number of Gramineae species also showed a significant negative correlation (*p* < .001; Figure 7a). In 2022, significant positive correlations were found between soil organic matter, total nitrogen and available phosphorus (*p* < .001; Figure 7b). Significant positive correlations were also detected between species richness and Margalef richness index, Shannon–Wiener diversity index, and Simpson dominance index (*p* < .001; Figure 7b).

**FIGURE 8:**
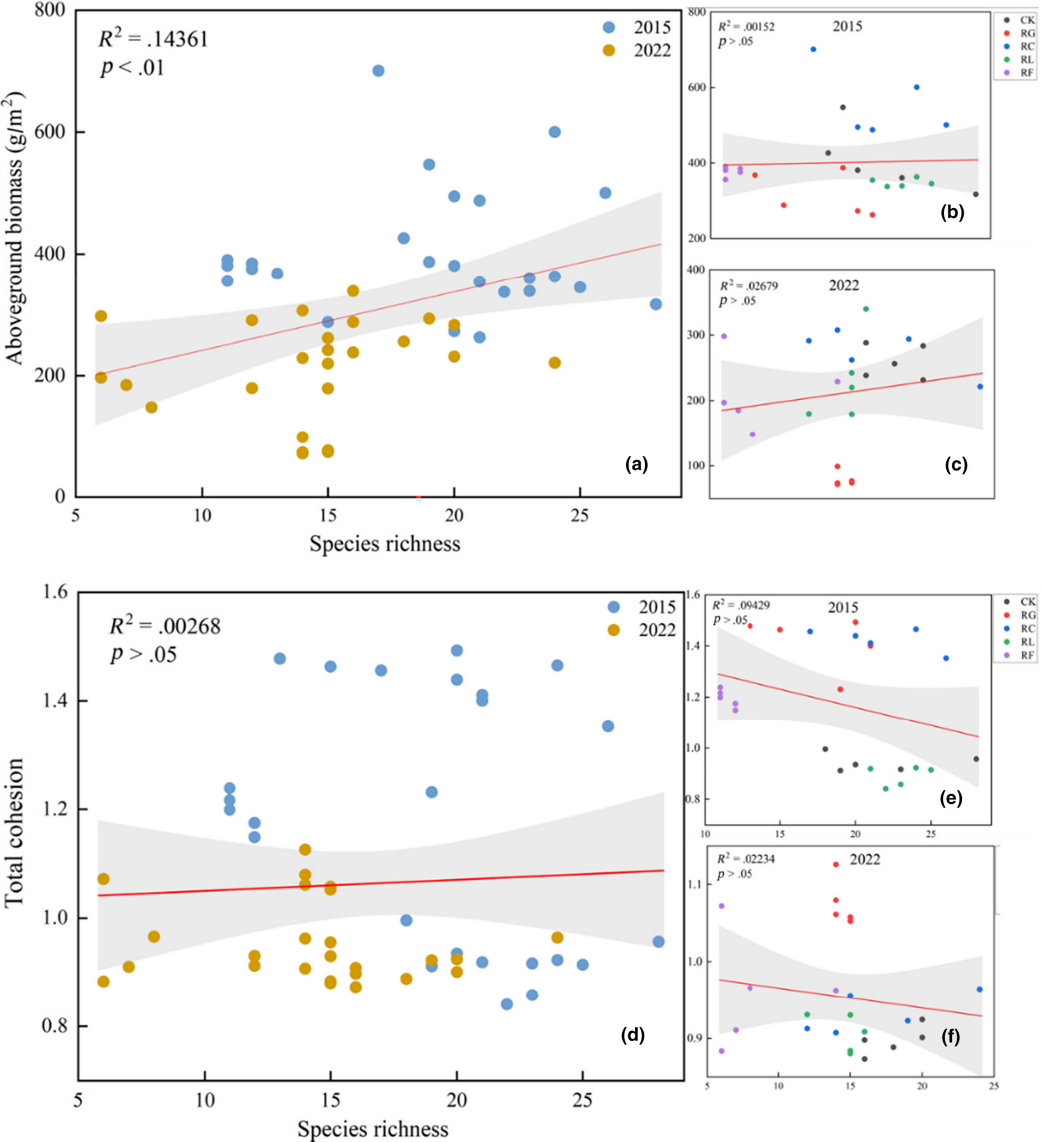
Relationships between species richness and plant productivity for (a) 2015 and 2022 pooled, and for (b) 2015 and (c) 2022 separately. Relationships between total cohesion and species richness for (d) 2015 and 2022 pooled, and for (e) 2015 and (f) 2022 separately. Gray shading represents 95% confidence intervals.

Overall, there was a significant positive correlation between the richness of species remaining in plant communities after treatment and aboveground biomass following both short‐ and long‐term PFG removal. Species diversity and biomass were not significantly correlated in 2015, but were significantly positively correlated in 2022, that is, after 10 years of treatment (Figure 8a–c). Total cohesion was not significantly positively correlated with species richness. Interestingly, short‐term PFG removal led to a stronger negative correlation between total cohesion and species richness for the remaining species compared to long‐term removal (Figure 8d–f).

## 4 DISCUSSION

We investigated the context dependence of the effects of species diversity loss on plant community structural composition and soil nutrients through long‐term PFG removal experiments at the local scale. In particular, we inspected the energy starvation hypothesis, in which removal behavior would lead to greater species loss effects through biomass reduction, and the niche complementarity hypothesis, in which target species removal would lead to greater changes in plant community structure and niche shifts among species (Figure 1). Contrary to both of these hypotheses, we found that partial treatment effects were relatively consistent and that most of these effects were related to the particular species removed and the species remaining within the community after treatment.

With prolonged time, the variation in species richness and above‐ and belowground biomass tended to gradually decrease, with nonsignificant differences in species richness in any treatments (Figure 3a–c; Table S1). Any removal generally reduced the number of Gramineae species remaining in the plant communities, and legume removal resulted in declines among all remaining species, such that species richness was reduced by an average of eight species (Figure 3d–g). Long‐term species loss generally led to declines in above‐ and belowground biomass, which was consistent with the energy starvation hypothesis and with previous findings that species removal significantly affects community productivity (Chen et al., 2016; Li et al., 2018). Despite the decline, there was no significant difference in species richness compared to the control, which is likely to be related to the compensatory effects hypothesis. In 2015, there were no significant differences in aboveground biomass among treatments where Cyperaceae, legumes, and other forbs were removed, perhaps because these communities were more sensitive to Gramineae removal (Figure 3a–g). Decreases in community productivity observed following any removal reflected declines in growth among the remaining plants in the community. For the other species remaining in the community, removal behavior had a larger effect on the Gramineae species, indicating that the Gramineae are less resistant to removal behavior; as reported in other studies, external influences are one of the key reasons for the decline in abundance and productivity of Gramineae (Dong et al., 2012).

Species diversity indices are used to effectively evaluate the heterogeneity and successional processes of plant communities. Our results showed a decreasing trend in the Margalef diversity index over time, whereas other indices showed inconsistent variation related to the numbers of Gramineae species in the treatments (Figure 4a–d); similar results were reported in a study of species diversity within plant communities (Jiang et al., 2003), and suggest that structural differences in communities dominated by different species can also lead to variation in diversity relationships. These findings highlight the importance of considering species abundance in determining plant diversity (Hooper & Vitousek, 1997).

In our study, species diversity and productivity had a positive relationship over time; a previous plant species removal experiment also showed a positive correlation between community productivity and species diversity, which remained consistent at the regional scale (Yang et al., 2004). Removal behavior not only decreased community species diversity but also reduced the productivity of different PFGs in the community (Tables S3–S5). The relationship between diversity and productivity is therefore likely to depend on community‐level productivity and species richness within various PFGs (Venail et al., 2010).

Soil carbon content is closely related to organic matter content and both characterize soil nutrient status. In most terrestrial ecosystems, N content limits vegetation productivity (LeBauer & Treseder, 2008). Total N, organic matter, and total P contents increased significantly in the legume removal treatment over time, while the content of available phosphorus and available nitrogen decreased. In the other treatments, soil nutrient contents were generally lower in 2022 than in 2015, available nitrogen changes irregularly (Figure 3h–n; Figure S1d–j). The number of species remaining in the community after removal of the target species decreased between 2015 and 2022, but aboveground biomass showed the smallest decrease in the legume removal treatment (Figure S1a–c; Tables S4 and S5), which demonstrated that the residual species are more sensitive to changes in soil nutrient content after legume removal (Symstad & Tilman, 2001). In contrast, because our treatments consisted of removing above‐ and belowground plant parts, it is possible that some legume roots remained in the soil, where N fixation may have continued through the symbiotic activity of rhizobia. Alternatively, the reduction of legume species may instead stimulate N fixation and other nutrient transformations in the remaining species, consistent with the niche complementarity hypothesis. Thus, the aboveground biomass of the plant community gradually decreased as the removal treatment continued from 2015 to 2022. As the biomass of the plant community gradually decreased with extended treatment, the N requirements of the community also decreased; these conditions could lead to increasing soil N content under further treatment extension (Bond, 1967; Mao, 2007). Thus, further research is needed to determine the response of legume species to extended PFG loss. N metabolism depends on carbon metabolism, which requires carbon resources and energy (Ayre, 2011; Lalonde et al., 2004; Udvardi & Poole, 2013; Yang et al., 2011), and carbon metabolism requires N metabolism to provide enzymes and photosynthetic pigments to cooperate with plant photosynthesis for N fixation (Evans & Clarke, 2019). Thus, an increase in organic matter content may be related to soil microorganism activity (Fanin et al., 2018). In this study, removal behavior tended to significantly affect nutrient accumulation in belowground plant parts; thus, the niche complementarity mechanism appeared to function differently for various plant, with the strongest effect observed following legume loss from the plant community.

We found that soil moisture content increased to varying degrees with prolonged removal treatment (Figure S1d). These results may be explained by increased rainfall due to climate change in recent years, or by community density and attenuated soil water evaporation effects; the exact mechanisms of these effects require further study. Soil moisture content greatly influences root nodule formation and N fixation activity. Most legumes form few root nodules or have low N fixation activity under drought conditions, whereas in moist soils, plants have high numbers of nodules, with larger volumes and higher N fixation activity (Bond, 1967). Compared to 2015, the legume treatment in 2022 increased total nitrogen but decreased available nitrogen, while other treatments increased available nitrogen (Figure S1f–j). Therefore, the increase in soil N observed in this study may be related to soil moisture content, and at the same time the legume loss may have stimulated the ability of other plants to fix nitrogen, so that the available nitrogen content of the legume treatment in the same year was not lower than that of other treatments. The total potassium content of the CK was significantly lower than that of the other treatments (Figure S1h). It was suggested that the removal behavior might have led to the increase in soil total potassium. The reason for this phenomenon may be that the removal of aboveground biomass results in plant death, and then potassium levels in the soil may increase as belowground plant parts decompose and mineralize potassium.

The result showed that different PFG removal treatments caused divergent changes in community cohesion. Total community cohesion was lower in 2022 than in 2015. The relative proportions of negative and positive cohesion were lower in the legume and other forb removal treatments in 2022 than in 2015, whereas the other treatments showed the opposite trend (Figure 5; Table 1). All treatments except the control had lower negative community cohesion in 2022 than in 2015, with the lowest value obtained in the other forb removal treatment. Positive species interactions can be driven by facilitation or mutually beneficial symbiotic relationships, whereas negative interactions may be driven by competition (Durán et al., 2018; Zelezniak et al., 2015). In the context of environmental filtering, positive correlations reflect niche or ecological function similarities among species (Chaffron et al., 2010), whereas species with diverse niches are negatively correlated (Hernandez et al., 2021). Moreover, community stability can be affected by the relative proportions of negative and positive associations (Coyte et al., 2015; Herren & McMahon, 2017; Mougi & Kondoh, 2012; Suweis et al., 2014).

In our study, PFG removal had an impact on plant community stability, such that the loss of some species caused niche changes among the remaining species; this niche divergence influenced the redistribution of community resources. The removal of forbs other than legumes led to the lowest negative cohesion values, indicating that the remaining species in this treatment had less stable interspecific associations, such that external perturbations made it difficult for the community to return to its previous equilibrium state (Agler et al., 2016; Coyte et al., 2015; Herren & McMahon, 2017). We also analyzed the relationship between total community cohesion and species richness; the correlation was indeed negative for a particular time period, but positive and insignificant from a combined perspective, suggesting the need for a longer exploratory analysis (Figure 5). NMDS analysis results showed the relationships among species remaining in the plant community after PFG removal, as well as niche status changes within the community (Figure 6). Following the removal of Gramineae, the plant community gradually developed from niche overlapping only for the other forb removal treatment in 2015 to overlapping among all other treatments in 2022; in contrast, the Cyperaceae removal treatment gradually changed from full overlap in 2015 to partial overlap in 2022, indicating that PFG removal altered the direction of community structure development.

The outcomes of our long‐term PFG removal experiments suggested that all impacts of species loss on ecosystem processes might be associated with species structural composition differences within the community. For example, legume removal increased soil organic matter and total N, whereas removing any species resulted in a significant reduction in the abundance of the remaining Gramineae species in the community. Several studies have shown that resource utilization, supplementarity, and facilitation regulate the effect of vegetation diversity on plant productive processes (Fridley, 2002; Wright et al., 2017). Our study also demonstrated that the impact of biodiversity loss on aboveground functions such as productivity might be strongly affected by the environmental context, and that soil communities might be driven by abiotic factors such as soil physicochemical properties, climatic disturbances, and parent material (Cameron et al., 2019; Delgado et al., 2019; Jucker et al., 2016; Ratcliffe et al., 2017; Sundqvist et al., 2014; Wardle et al., 2012, 2013). The mechanism by which long‐term species loss at the local scale contributes to niche shifts among the remaining species within the community remains to be explored, including the dependence of these effects on the spatial scale and whether they result in the loss of multiple ecosystem functions relevant to the specific PFGs removed from the community.

## 5 CONCLUSIONS

Our results indicated that the influence of species diversity loss on ecosystems may be inextricably linked to differences in the structural composition of species within communities. After 3 and 10 years of treatment, the removal of different PFGs prompted responses to both energy starvation and niche complementarity to some extent, which was associated with species removal and structural composition differences within the community, respectively. More importantly, all PFG removal treatments resulted in a significant reduction of Gramineae within the plant community, indicating that Gramineae are less resistant to removal, which is a key factor influencing decreases in community richness and productivity. Unexpectedly, the removal of legume species led to significant increases in soil moisture and nutrient contents. This finding is presumably mainly due to the unique root physiology of legumes, but likely also indirectly reflects differences in the niche complementarity among different PFGs. Our analysis of community cohesion in response to PFG removal demonstrated that the forb removal other than legumes made it difficult for the community to return to its previous equilibrium state within a short time. Consequently, community‐level species loss led to niche shifts among individual species, prompting a redistribution of community resources. Therefore, we emphasize the need for continued long‐term PFG removal experiments and studies that examine a wider range of domains at broader spatial and temporal scales, to obtain a more accurate and refined response mechanism.

## AUTHOR CONTRIBUTIONS

**Jingjing Wei:** Data curation (equal); methodology (equal); project administration (equal); resources (equal); software (equal); supervision (equal); validation (equal); visualization (equal); writing – original draft (equal); writing – review and editing (equal). **Huakun Zhou:** Funding acquisition (equal); resources (equal); writing – review and editing (equal). **Xinqing Shao:** Software (equal). **Jian Sun:** Resources (equal). **Li Ma:** Conceptualization (lead). **Zhonghua Zhang:** Methodology (equal). **Ruimin Qin:** Formal analysis (equal). **Hongye Su:** Methodology (equal). **Xue Hu:** Data curation (equal). **Tao Chang:** Investigation (equal). **Zhengchen Shi:** Formal analysis (equal). **Haze Ade:** Data curation (equal). **Huichun Wang:** Formal analysis (equal).

## CONFLICT OF INTEREST STATEMENT

The authors declare that they have no known competing financial interests or personal relationships that could have appeared to influence the work reported in this paper. In addition, the publication is approved by all authors.

## Supporting information


Appendix S1.

## Data Availability

Data will be made available on request.
