# Supplementary material for: Effects of short‐ and long‐term plant functional group loss on alpine meadow community structure and soil nutrients
Source: Ecol Evol. 2024 Mar 11;14(3):e10919. doi: 10.1002/ece3.10919 (PMC10928257; doi:10.1002/ece3.10919)
Supplement: Supplementary file 1 — Appendix S1. [file ECE3-14-e10919-s001.docx]

**Appendix A. Supplementary data**

**TABLE S1** The F and P values from the two-way ANOVA for species (Species richness, Aboveground biomass, Belowground biomass, Number of Gramineae, Number of Cyperaceae, Number of legumes and Number of forbs) affected by year and removal type (CK, RG, RC, RL and RF)

| Source | Species  richness | | | Aboveground biomass | | Belowground biomass | | Number of Gramineae | | Number of Cyperaceae | | Number of  legumes | | Number of  forbs | |
| --- | --- | --- | --- | --- | --- | --- | --- | --- | --- | --- | --- | --- | --- | --- | --- |
|  | df | F | *P* | F | *P* | F | *P* | F | *P* | F | *P* | F | *P* | F | *P* |
| Year | 1 | 0.93 | 0.335 | 153.0 | <0.0001 | 79.08 | <0.0001 | 26.94 | <0.0001 | 26.60 | <0.0001 | 18.84 | <0.0001 | 85.16 | <0.0001 |
| Treatment | 4 | 4.62 | 0.004 | 21.28 | <0.0001 | 2.22 | 0.084 | 99.56 | <0.0001 | 2.32 | 0.135 | 0.028 | 0.868 | 5.11 | 0.029 |
| Year*Treatment | 4 | 13.45 | <0.0001 | 3.94 | 0.009 | 2.37 | 0.068 | 7.70 | <0.0001 | 1.33 | 0.276 | 0.310 | 0.870 | 2.18 | 0.089 |

**TABLE S2** The F and P values from the two-way ANOVA for soil properties (SM, SOM, TP, TN, AP, AN and TK ) affected by year and removal type (CK, RG, RC, RL and RF)

| Source | SM | | | SOM | | TP | | TN | | AP | | AN | | TK | |
| --- | --- | --- | --- | --- | --- | --- | --- | --- | --- | --- | --- | --- | --- | --- | --- |
|  | df | F | *P* | F | *P* | F | *P* | F | *P* | F | *P* | F | *P* | F | *P* |
| Year | 1 | 57.31 | <0.0001 | 2.92 | 0.095 | 0.65 | 0.426 | 1.84 | 0.183 | 928.27 | <0.0001 | 1.94 | 0.170 | 0.43 | 0.514 |
| Treatment | 4 | 4.73 | 0.003 | 3.95 | 0.009 | 1.82 | 0.143 | 5.57 | 0.001 | 25.65 | <0.0001 | 3.83 | 0.010 | 3.61 | 0.013 |
| Year*Treatment | 4 | 2.88 | 0.034 | 8.48 | <0.0001 | 10.40 | <0.0001 | 4.08 | 0.007 | 33.64 | <0.0001 | 3.35 | 0.019 | 0.42 | 0.796 |

**TABLE S3** Number of plant functional groups species in 2015 vs. 2022.

| Plant functional groups | 2015 | | | | | 2022 | | | | |
| --- | --- | --- | --- | --- | --- | --- | --- | --- | --- | --- |
|  | CK | RG | RC | RL | RF | CK | RG | RC | RL | RF |
| Total number of species | 21.6 | 17.6 | 21.6 | 23 | 11.4 | 18 | 14.4 | 16.8 | 14.6 | 8.2 |
| Number of Gramineae | 5.6 | 0 | 5.4 | 6 | 5.6 | 1.2 | 0 | 2.6 | 1.4 | 2.6 |
| Number of Cyperaceae | 2.8 | 2.8 | 0 | 3.4 | 2.8 | 2.4 | 2.6 | 0 | 2.2 | 2.8 |
| Number of legumes | 2.4 | 2.8 | 2.8 | 0 | 2.4 | 2.4 | 2.4 | 2.6 | 0 | 2.8 |
| Number of forbs | 10.8 | 12 | 13.4 | 13.6 | 0 | 12 | 9.4 | 11.6 | 11 | 0 |

Note: CK, RG, RC, RL and RF represent no-removal control, removal of grass, removal of Cyperaceae, removal oflegumes, removal of forbs.

**TABLE S4** Biomass of plant functional groups species in 2015

| PFGs biomass | CK (g·m^-2^) | RG (g·m^-2^) | RC (g·m^-2^) | RL (g·m^-2^) | RF (g·m^-2^) |
| --- | --- | --- | --- | --- | --- |
| Community biomass | 406.52±87.88 | 315.93±57.47 | 532.16±75.20 | 348.11±32.37 | 375.86±77.49 |
| Gramineae biomass | 234.84±84.97 | / | 390.04±63.15 | 158.38±70.54 | 275.96±97.57 |
| Cyperaceae biomass | 31.60±19.77 | 57.21±26.00 | / | 52.24±20.34 | 65.54±34.36 |
| legumes biomass | 16.35±13.78 | 38.16±22.55 | 32.56±15.14 | / | 34.36±4.69 |
| forbs biomass | 93.85±57.16 | 220.56±57.95 | 109.56±75.73 | 137.49±51.93 | / |

**TABLE S5** Biomass of plant functional groups species in 2022

| PFGs biomass | CK (g·m^-2^) | RG (g·m^-2^) | RC (g·m^-2^) | RL (g·m^-2^) | RF (g·m^-2^) |
| --- | --- | --- | --- | --- | --- |
| Community biomass | 259.54±25.62 | 79.14±11.19 | 275.14±34.26 | 232.14±65.76 | 211.45±56.35 |
| Gramineae biomass | 191.37±33.01 | / | 193.32±45.27 | 165.58±67.95 | 4.00±1.50 |
| Cyperaceae biomass | 3.79±6.64 | 12.03±5.6 | / | 4.08±3.52 | 198.84±55.61 |
| legumes biomass | 2.42±1.74 | 2.21±1.9 | 1.87±1.08 | / | 9.40±3.21 |
| forbs biomass | 61.95±25.34 | 64.89±23.87 | 79.94±22.34 | 63.29±24.81 | / |


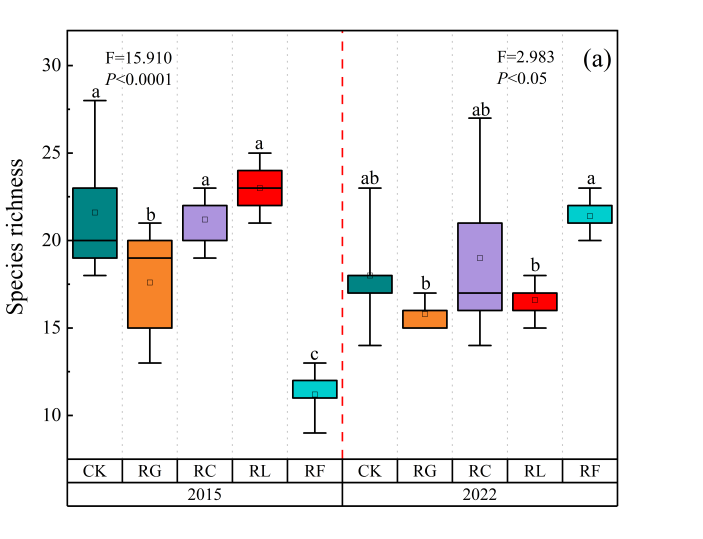

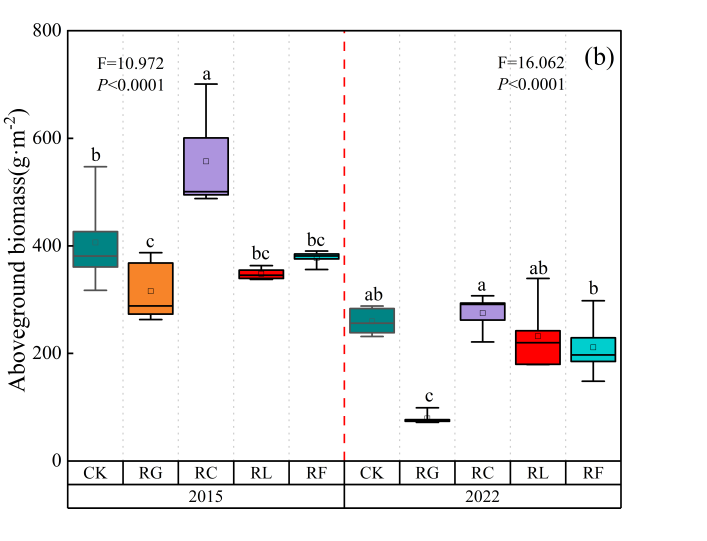


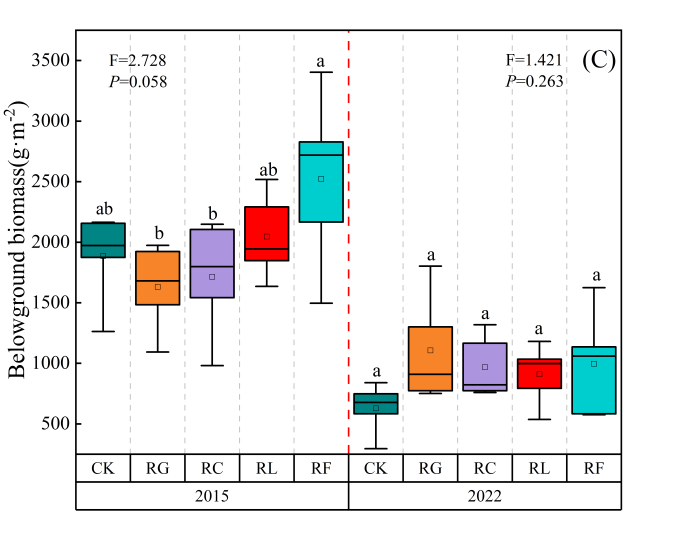

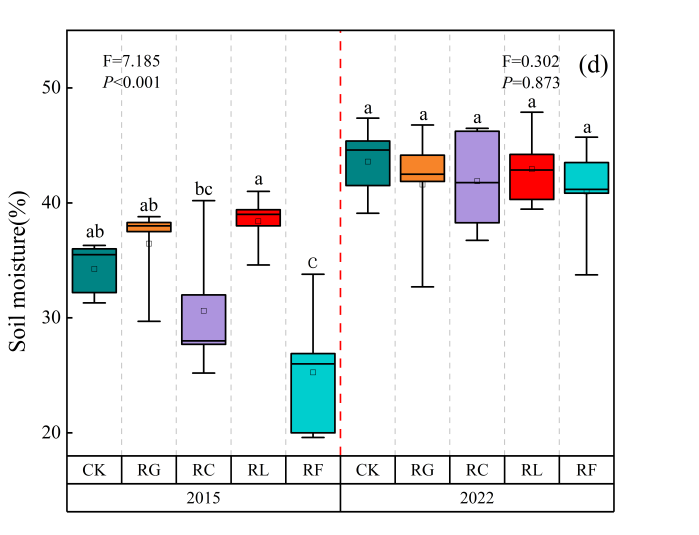


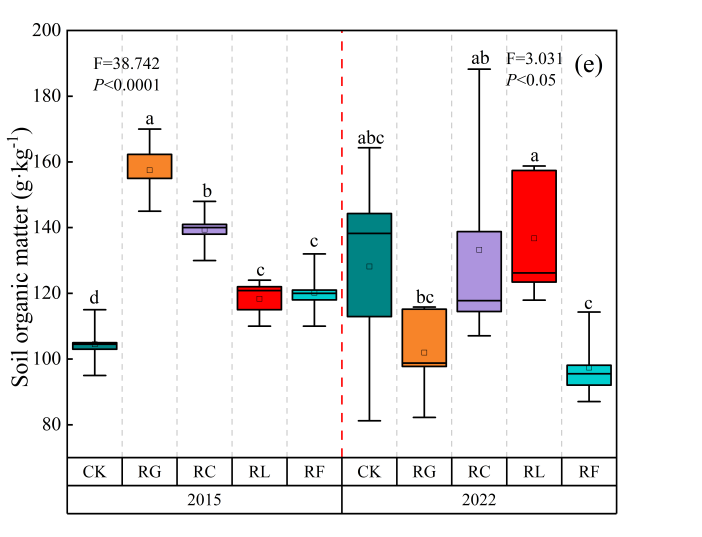

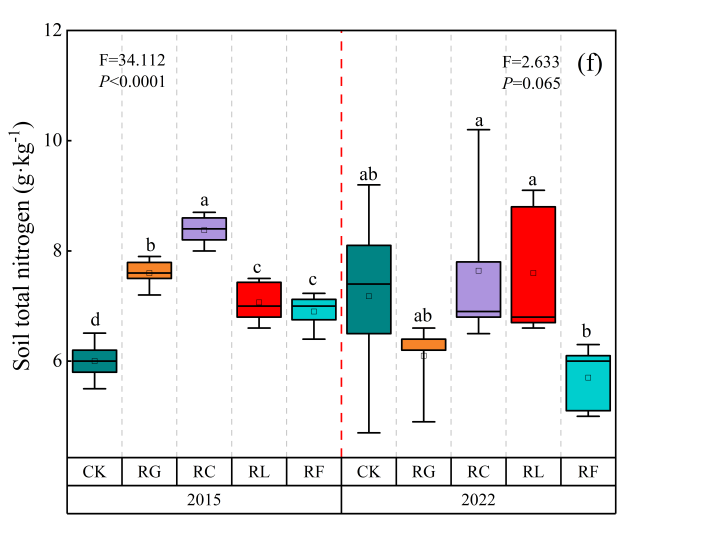


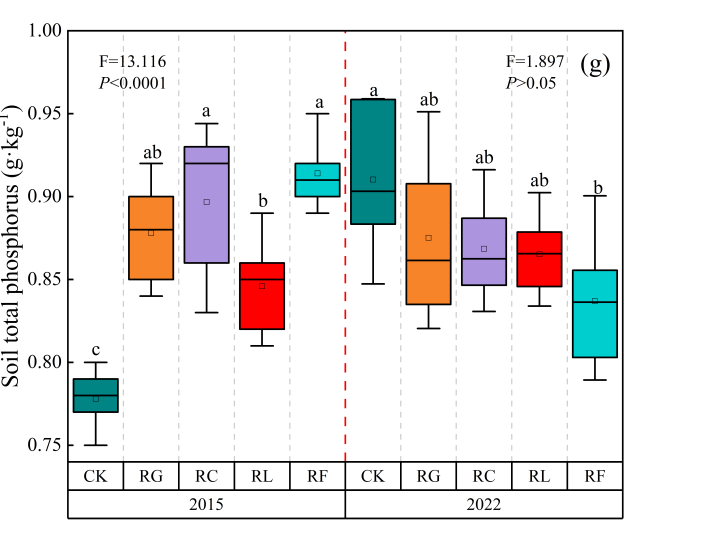

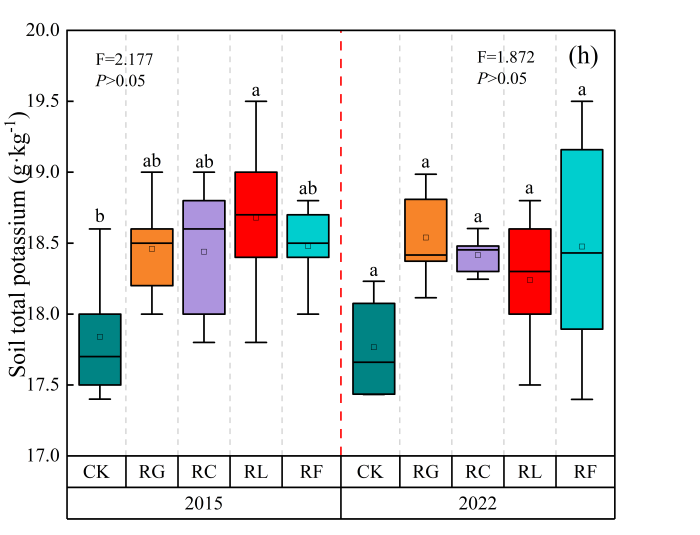


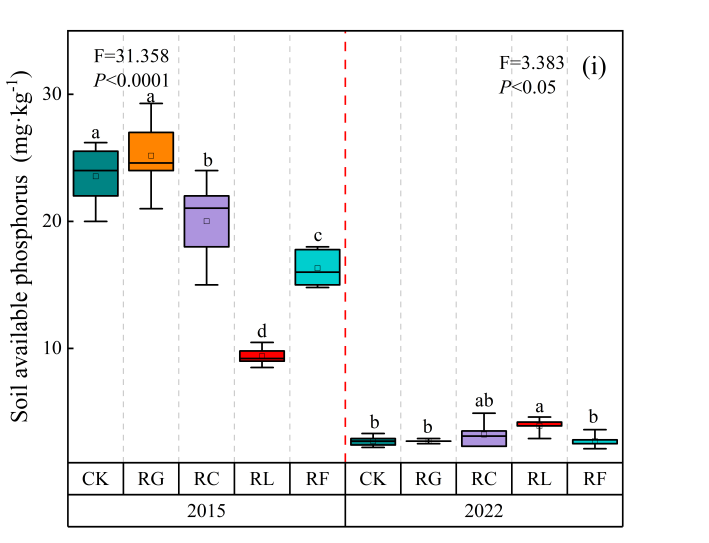

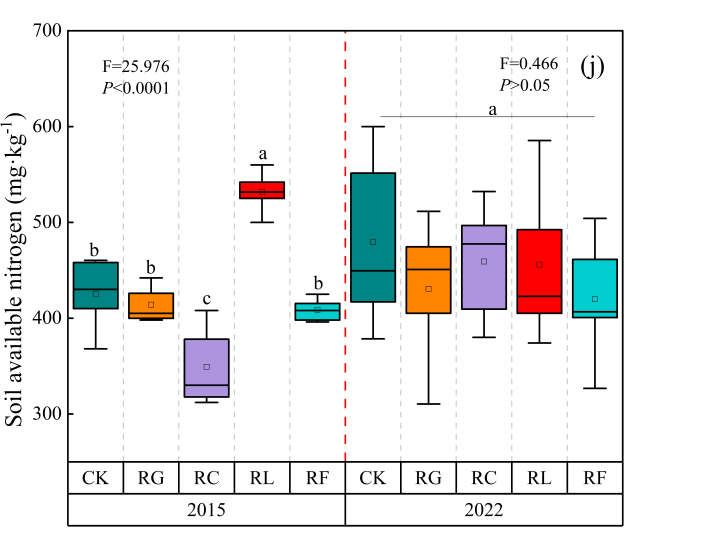

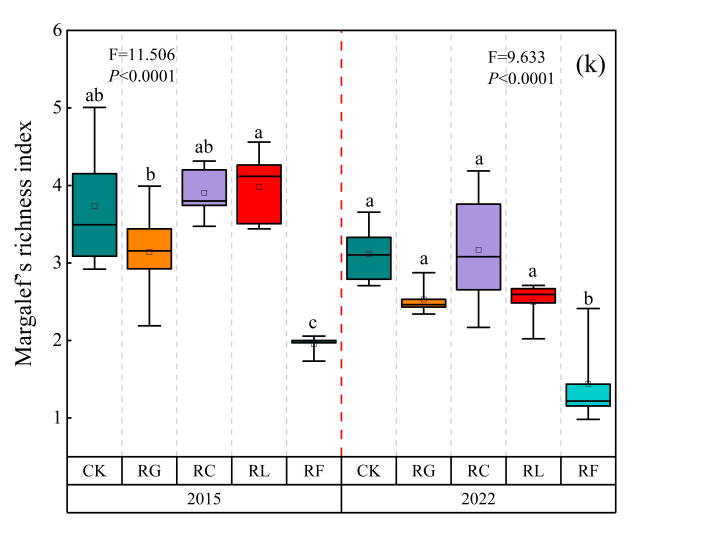

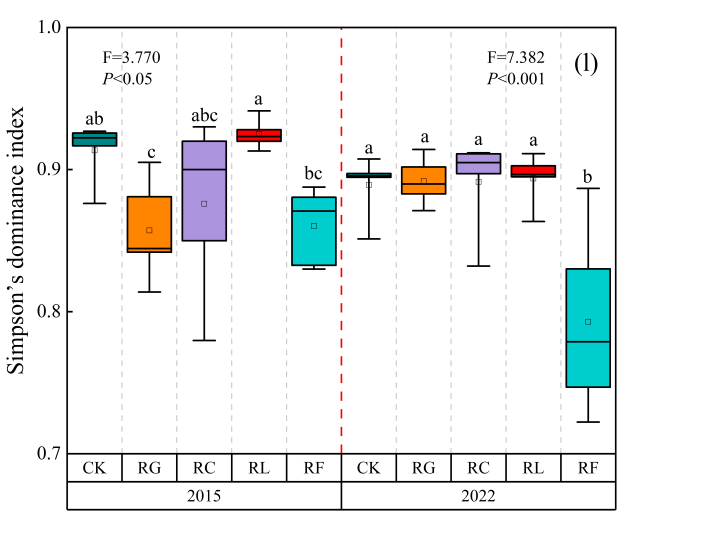


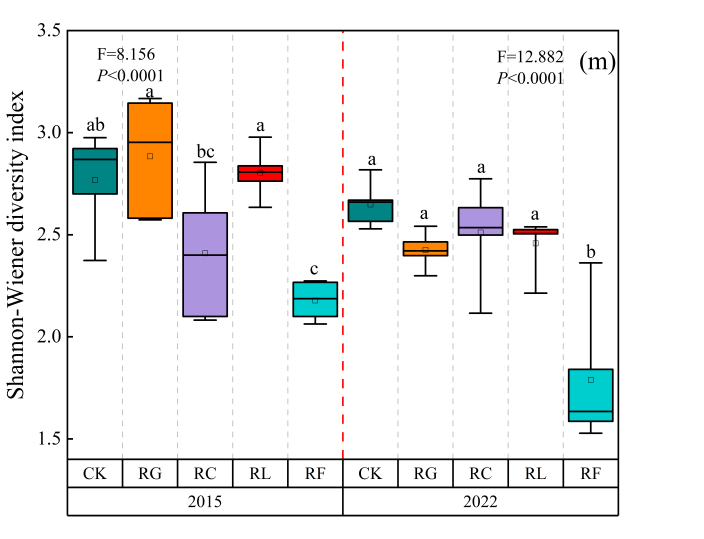

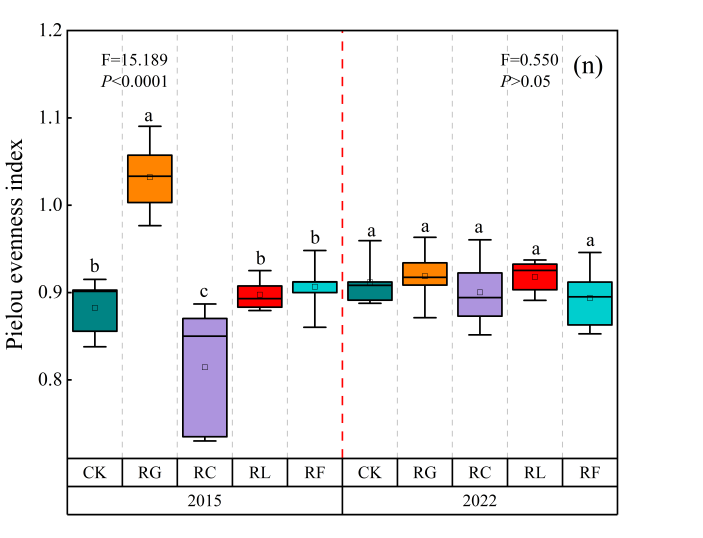


**FIGURE S1** Species richness (a), Aboveground biomass (b), Belowground biomass (c), Soil moisture (d), Soil organic matter (e), Soil total nitrogen (f), Soil total phosphorus (g), Soil total potassium (h), Soil available phosphorous (i), Soil available nitrogen (j), Margalef richness index (k), Simpson dominance index (l), Shannon–Wiener diversity index (m), Pielou evenness index (n) to PFGs in 2015 and 2022. The error bars represent the 95% confidence interval. Different lowercase letters represent the significant differences among different treatment types. CK, RG, RC, RL and RF represent no-removal control, removal of Gramineae, removal of Cyperaceae, removal of legumes, removal of forbs.


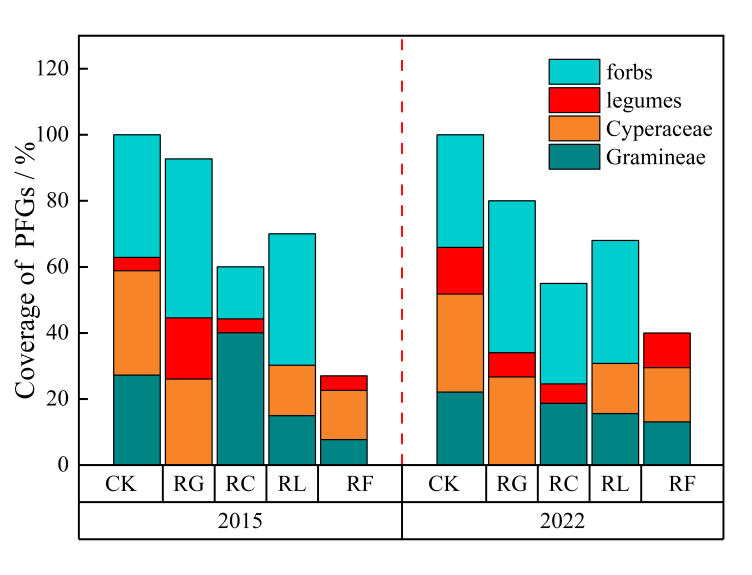


**FIGURE S2** Coverage of PFGs in 2015 and 2022. CK, RG, RC, RL and RF represent no-removal control, removal of Gramineae, removal of Cyperaceae, removal of legumes, removal of forbs.
